# ^1^H, ^13^C and ^15^N resonance assignments for the tandem CUE domains from chromatin remodeler SMARCAD1

**DOI:** 10.1007/s12104-019-09888-9

**Published:** 2019-03-27

**Authors:** Antonio J. Biasutto, Philip M. West, Erika J. Mancini, Christina Redfield

**Affiliations:** 1 Department of Biochemistry, University of Oxford, South Parks Road, Oxford, OX1 3QU UK; 2 Division of Structural Biology, Henry Wellcome Building for Genomic Medicine, University of Oxford, Roosevelt Drive, Oxford, OX3 7BN UK; 3 School of Life Sciences, Biomedicine and Biochemistry Department, University of Sussex, Brighton, BN1 9QG UK

**Keywords:** CUE domain, SMARCAD1, Chromatin remodelling, ATPase, NMR resonance assignments, Secondary structure

## Abstract

SMARCAD1 is a non-canonical chromatin remodelling ATPase, unique in its domain organization in that is encodes tandem ubiquitin binding CUE domains along with a classical SNF2 helicase ATP-dependent motor. SMARCAD1 is conserved from yeast to humans and has reported roles in the maintenance of heterochromatin following replication and in double-strand break repair. Here we present the ^1^H, ^13^C and ^15^N assignments for the tandem CUE domains and for the disordered regions that flank them. These assignments provide the starting point for detailed investigations of the structure and interactions of this region of SMARCAD1.

## Biological context

SMARCAD1 (also known as Etl-1, HEL-1 or KIAA1122) is a non-canonical member of the SNF2 family of chromatin remodelling ATPases, unique in its domain organization encoding two CUE domains (previously characterized as mono-ubiquitin binding domains) along with a classical SNF2 helicase ATP-dependent motor (Okazaki et al. 2008; Schoor et al. 1999; Soininen et al. 1992). SMARCAD1 belongs to the Swr1-like subfamily, which is evolutionarily the most conserved class of chromatin remodelling ATPases, from yeast to humans. SMARCAD1 has been shown to play many key cellular roles, some of which are functional extrapolations from the roles of the well-characterised *S. cerevisiae* ortholog FUN30 (Chen et al. 2012; Eapen et al. 2012). SMARCAD1, in association with PCNA and transcriptional repressors KAP1, histone deacetylases HDAC1/2 and histone methyltransferase G9a/GLP, is a key factor required for the re-establishment of repressive chromatin structures following replication (Mermoud et al. 2011; Rowbotham et al. 2011). Conversely, together with co-activator p300/CBP SMARCAD1 can activate transcription (Doiguchi et al. 2016). Recently, SMARCAD1 has been linked to double-strand break (DSB) resection in homologous recombination (HR) (Costelloe et al. 2012; Densham et al. 2016). Specifically, the tandem CUE domains are thought to be required for binding ubiquitinated histone H2A (H2A-Ub) at the sites of DNA damage and for the eviction of tumour suppressor p53-binding protein 1 (53BP1) to ensure complete resection of DSBs during HR. However, the molecular mechanisms through which SMARCAD1 exercises these key cellular processes are poorly understood and possible roles for its CUE domains have not been elucidated.

Secondary structure prediction of the SMARCAD1 sequence suggests that the N-terminal tandem CUE domains, termed CUE1 (residues 157–199) and CUE2 (residues 251–294), are each composed of a three helix bundle and are connected by a disordered linker rich in serines and charged amino acids (residues 200–250) (Neves-Costa et al. 2009). Functionally, the members of the CUE domain family have been characterized as mono-ubiquitin binding domains; however, sequence conservation is low both between CUE1 and CUE2 and between SMARCAD1 CUE domains and canonical members of the CUE domain family, such as yeast Cue2 or human TOLLIP (Dikic et al. 2009; Hurley et al. 2006). Specifically, sequence conservation is low amongst key residues involved in ubiquitin recognition suggesting a reduced affinity for the canonical substrate.

To gain an understanding of the molecular mechanism by which SMARCAD1 employs its CUE domains to establish protein–protein interactions, we have embarked on an NMR spectroscopy study to characterise their structure and dynamics. Here we present the ^1^H, ^13^C and ^15^N assignments for two overlapping constructs derived from this region of SMARCAD1. The first, dCUE, corresponds to the tandem CUE domains spanning residues 144–295. The second, eCUE, corresponds to only the first CUE domain and the disordered region preceding it (residues 109–206).

## Methods and experiments

### Protein expression and purification

Truncation constructs corresponding to dCUE and eCUE were amplified using full-length human SMARCAD1 cDNA (IOH26772, Invitrogen ORF collection—kindly donated by Dr Patrick Varga-Weisz) by PCR using an appropriate set of primers (Table 1), cloned into plasmids bearing a T7 promoter using restriction enzyme or ligation independent cloning, and encoding a hexahistidine tag. Recombinant fusion proteins were expressed in Rosetta2 (DE3) pLysS or pLacI depending on the specific construct vector.

**Table 1:** SMARCAD1 truncation constructs

Construct	SMARCAD1 region	Primer pairs	Expression vector
dCUE	144–295	5′cgggatccggatatttcagaactggaaga3′ 5′ccttaaggctagtcttctgcaaacactttta3′	pET-DUET-1
eCUE	109–206	5′aggagatataccatgacagttcaagagaaaacattcaacaaagatacagtg3′ 5′gtgatggtgatgtttccatttcctgggcccaccacctg3′	pOPINE

^15^N-single-labelled and ^15^N/^13^C-double-labelled proteins were produced in M9 minimal media containing ^15^N–NH_4_Cl (1 g/L) (Sigma-Aldrich) or ^13^C_6_-glucose (4 g/L) (Cambridge Isotope Laboratories) as sole nitrogen and carbon sources, respectively. Freshly transformed colonies were used to inoculate a small-scale (~ 15 mL) LB culture supplemented with 50 µg/mL of carbenicillin and 35 µg/mL chloramphenicol, which was incubated overnight at 37 °C while shaking at 160 rpm. The pellet collected after briefly centrifuging at 4500 ×*g* was resuspended in a medium-scale (~ 150 mL) culture of M9 minimal media with the appropriate isotopically enriched nitrogen and carbon sources according to the desired labelled strategy and supplemented with 50 µg/mL of carbenicillin and 35 µg/mL chloramphenicol. The culture was incubated at 30 °C overnight while shaking at 180–220 rpm, and later used to inoculate a large-scale culture of minimal media for expression in volumes up to 20% of the total Erlenmeyer flask volume (normally 400 mL in 2L flasks), with an initial optical density (OD) (λ = 600 nm) of ~ 0.06–0.08. The flasks were shaken at 180–220 rpm at 37 °C until OD (λ = 600 nm) was between 0.6 and 0.8, cooled to 18 °C and induced with 1 mM IPTG (Melford) while shaking for a further 12–16 h. Cultures were harvested by centrifugation at 4500 ×*g* for 30 min before discarding of waste media; the remaining pellets were rinsed once with phosphate buffered saline (Sigma-Aldrich) and stored at − 20 °C. Expression of individual culture batches was checked by harvesting a 1 mL aliquot of each culture and verifying the total cell lysate protein pattern by SDS-PAGE.

Bacterial cell pellets stored at − 20 °C were thawed on ice and resuspended to ~ 0.1 g/mL in low salt HisTrap binding buffer (50 mM Tris HCl pH 8.0, 150 mM NaCl, 20 mM imidazole pH 8.0) supplemented with complete EDTA-free protease inhibitor cocktail (Roche), 80 U/mL DNAse I (Sigma) and 0.25 mg/mL lysozyme (Sigma). After homogenizing the solution while stirring at 4 °C, cells were lysed via cell disruption (2 passes, 26kpsi) and cleared through centrifugation at 4 °C and 48,000 ×*g* for 1 h; the supernatant was then filtered through a 0.45 µm syringe filter (Millipore) and loaded on a pre-equilibrated 5 mL HisTrap HP column (GE Healthcare) using an ÄKTA Purifier system (GE Healthcare). The column was washed with five column volumes (CV) of binding buffer and all bound proteins were eluted over a 10CV linear gradient to 500 mM imidazole pH 8.0. All fractions were monitored by their absorption at 280 nm/260 nm, collected and their composition assessed by SDS-PAGE. Fractions containing the protein of interest were pooled and diluted 1:3 with 50 mM Tris HCl pH 8.0 and loaded on to a pre-equilibrated 5 mL HiTrap Q HP ion-exchange column (GE Healthcare) using an ÄKTA purifier system. Unbound material was washed off with 5CV of low salt IEX-Q binding buffer (50 mM Tris HCl pH 8.0, 50 mM NaCl), which was followed by a linear 10CV gradient to 1M NaCl for elution of bound proteins. All fractions were collected and their composition determined by SDS-PAGE. Fractions containing the protein of interest were pooled and concentrated using an Amicon centrifugation filter device (Millipore) with molecular weight cut-off between 3 kDa and 50 kDa, at 4500 ×*g* and 4 °C. The concentrated protein solution was applied at 1 mL/min to a HiLoad 16/600 Superdex 75 size-exclusion column (GE Healthcare) equilibrated with SEC Phosphate Buffer (20 mM NaH_2_PO_4_ pH 7.0 and 100 mM NaCl). Appropriate fractions, identified by SDS-PAGE analysis, were pooled, concentrated with an Amicon centrifugation filter and stored at − 80 °C until further use.

### NMR spectroscopy

^15^N or ^15^N/^13^C-double-labelled samples of dCUE and eCUE were used for resonance assignment using standard protocols (Redfield 2015). All samples contained 95% H_2_O/5% D_2_O (v/v) in a 20 mM NaH_2_PO_4_ pH 7.0 buffer with 100 mM NaCl. NMR experiments were carried out at 293 K using 500, 600 and 750 MHz spectrometers. The 500 and 600 MHz spectrometers were equipped with Bruker Avance consoles and TCI CryoProbes. The 750 MHz spectrometer was equipped with a home-built console and room-temperature triple-resonance probe.

Resonance assignments for dCUE were obtained using 2D ^1^H–^13^C and ^1^H–^15^N HSQC experiments and 3D NMR experiments including ^15^N-edited NOESY-HSQC, ^15^N-edited TOCSY-HSQC, HNCA, HN(CO)CA, CBCANH, CBCA(CO)NH, HNCO, HN(CA)CO, HBHA(CBCACO)NH, (H)C(CCO)NH, and HCCH-TOCSY. The ^15^N-edited experiments and the HCCH-TOCSY were collected at 750 MHz and the triple-resonance experiments were collected at 500 MHz. Resonance assignments for eCUE were obtained using 2D ^1^H–^13^C and ^1^H–^15^N HSQC experiments and 3D NMR experiments including ^15^N-edited NOESY-HSQC, ^15^N-edited TOCSY-HSQC, CBCANH, CBCA(CO)NH, HNCO, HBHA(CBCACO)NH, H(CCCO)NH, (H)C(CCO)NH and HCCH-TOCSY. The eCUE data were collected at 600 MHz. Details of the specific experiments used for each of the sample conditions can be found in the BMRB deposition files.

NMR data were processed using NMRPipe (Delaglio et al. 1995) and analysed using CcpNmr Analysis (Vranken et al. 2005). ^1^H chemical shifts were referenced using the H_2_O peak (4.8 ppm at 293 K), previously calibrated with DSS, and ^13^C and ^15^N were referenced indirectly.

## Extent of assignments and data deposition

**Fig. 1:**
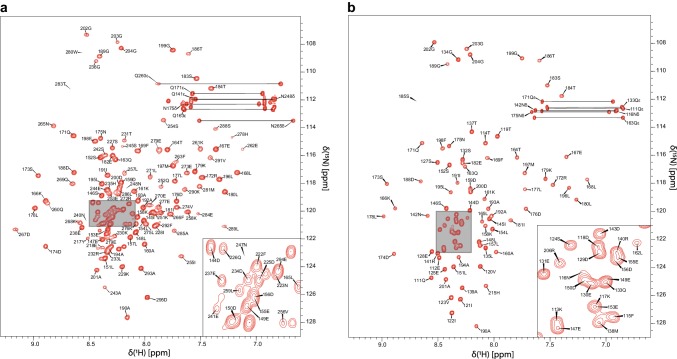
**a** 500 MHz ^1^H–^15^N HSQC spectrum of dCUE in 20 mM sodium phosphate, 100 mM NaCl (95% H_2_O/5% D_2_O), at pH 7.0, 293 K. **b** 600 MHz ^1^H–^15^N HSQC spectrum of eCUE in 20 mM sodium phosphate, 100 mM NaCl (95% H_2_O/5% D_2_O), at pH 7.0, 293 K. The peak assignments for backbone amides are annotated. Non-degenerate protons of the side chain amino groups are connected by a line. The inset corresponds to a magnification of the shaded area on the spectrum

Figure 1 shows the ^1^H–^15^N HSQC spectra of dCUE and eCUE at pH 7.0. ^1^H^N^ and ^15^N backbone resonances for 127 of the 148 non-proline residues of dCUE and for 91 of the 94 non-proline residues of eCUE were assigned. ^1^H^N^ and ^15^N chemical shifts for dCUE residues L162, S185, M187, R206—E215, E221, D224, S239, S246, W249, E250, Y282, and E287 were not assigned. Similarly, ^1^H^N^ and ^15^N assignments for eCUE residues T109, V110, and M187 were not obtained. The assignment statistics for dCUE and eCUE are summarised in Table 2. Moreover, the statistics for dCUE are reported for the individual functional domains, from which it is evident that the relatively poor overall assignment coverage stems from the flexible linker connecting the two CUE domains. This is mostly due to high sequence degeneracy and the lack of structural propensity in this region of the protein.

**Table 2:** Extent of assignment for dCUE and eCUE

Sample	Percent assigned				
	^1^H^N^/^15^N^a^	^13^Cʹ	^1^Hα/^13^Cα	^1^Hβ/^13^Cβ	^1^Hγ/^13^Cγ^b^
dCUE	85.8/85.8	86.8	89.2/92.8	85.9/92.5	55.7/60.4
CUE1	92.9/92.9	100	100/100	100/100	71.4/78.6
Linker	67.3/67.3	64.7	70.9/78.4	60.0/76.6	43.2/45.8
CUE2	95.3/95.3	95.5	100/100	97.5/100	51.7/55.6
eCUE	96.8/96.8	91.8	99.0/99.0	99.4/98.9	98.1/98.5

^a^Assignment statistics are for residues of the native sequence. The nitrogens for the proline residues are not included in the statistics

^b^Gamma carbons from Asp, Asn, His, Phe, Tyr and Trp, which do not have attached ^1^H and are generally not assigned, are not included in the statistics

**Fig. 2:**
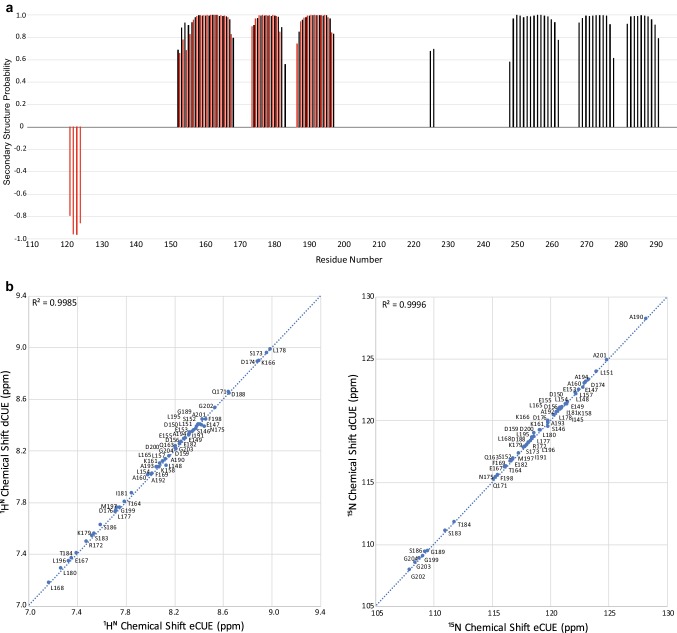
**a** Secondary structure probabilities (SSPs), calculated from the ^13^Cα, ^13^Cβ, ^13^C´, ^1^Hα, ^1^H^N^ and ^15^N chemical shifts using TALOS-N are plotted as a function of amino acid sequence for dCUE (Black) and eCUE (Red) at pH 7.0. Positive and negative SSPs are indicative of α-helix and β-sheet structure, respectively. **b** Correlation between secondary chemical shifts of the common residues in dCUE and eCUE for ^1^H^N^ (left) and ^15^N (right) showing no changes for the chemical shifts of the shared CUE1 domain between the two protein constructs studied here

The ^13^Cα, ^13^Cβ, ^13^Cʹ ^1^Hα, ^1^H^N^ and ^15^N chemical shifts have been used to predict secondary structure propensities for dCUE and eCUE in solution using TALOS-N (Shen and Bax 2015); these are plotted as a function of sequence in Fig. 2a. The predicted secondary structure shows the expected pattern of three α-helices in each CUE domain. Interestingly, a short β-strand is predicted in the otherwise disordered region preceding CUE1.

No significant changes in chemical shift are observed in the ^1^H–^15^N HSQC for the residues of the CUE1 domain in the dCUE and eCUE constructs (Fig. 2b). This suggests that the N-terminal region, the linker, and the CUE2 domain do not make specific contacts with the CUE1 domain.

The chemical shift assignments for dCUE and eCUE at pH 7.0 have been deposited in the BioMagResBank (http://www.bmrb.wisc.edu) under the accession numbers 27785 and 27780, respectively.
